# Supplementary material for: Longitudinal relationships between habitual physical activity and pain tolerance in the general population
Source: PLoS One. 2023 May 24;18(5):e0285041. doi: 10.1371/journal.pone.0285041 (PMC10208467; doi:10.1371/journal.pone.0285041)
Supplement: S1 Table — The Tromsø Study 2007–2016. (DOCX) [file pone.0285041.s001.docx]

| Table S1: Primary analysis sample missing data on baseline covariates (N=6,864). The Tromsø Study 2007-2016. | |
| --- | --- |
| Covariable: | **n (%)** |
| Sex | 0 (0) |
| Age | 0 (0) |
| Education level | 38 (0.5) |
| Alcohol consumption frequency | 28 (0.4) |
| Self-reported health | 49 (0.7) |
| Daily smoking status | 54 (0.8) |
| Chronic pain | 6 (0.1) |
| Occupational PA/retired/sick leave/disability | 87 (1.3) |
| PA= physical activity. | |
