# Supplementary material for: Longitudinal relationships between habitual physical activity and pain tolerance in the general population
Source: PLoS One. 2023 May 24;18(5):e0285041. doi: 10.1371/journal.pone.0285041 (PMC10208467; doi:10.1371/journal.pone.0285041)
Supplement: S3 Table — The Tromsø Study 2007–2016. Mixed model Tobit regression with upper limit (censoring) = 106 s. for latent distribution of CPT outcome. Models were adjusted for measurement occasion, as well as baseline sex, age, and self-reported occupational PA level, education, alcohol consumption frequency, smoking status, health status, and chronic pain. Significant results in bold. (DOCX) [file pone.0285041.s003.docx]

| Table S3: Regression coefficients with 95% confidence limits for the association between baseline levels of leisure-time physical activity and cold-pressor tolerance time (seconds) by sex or chronic pain^a^. The Tromsø Study 2007-2016. | | | | |
| --- | --- | --- | --- | --- |
|  | |  | | |
| **LTPA** | **n=10,254** | | **Sub-groups ^b^** | |
| Reference group CPT tolerance**^c^** | 1,962 | | Female | Male |
|  |  |  | *90.4 (86.4, 94.5)* | *116.9 (114.3, 119.6)* |
| LTPA |  | |  |  |
| *Sedentary* | 1,962 | | 0 (reference) | 0 (reference) |
| *Light* | 6,178 | | **5.0 (0.5, 9.4**) | **8.5 (3.7, 13.2)** |
| *Moderate* | 1,933 | | **10.1 (3.9, 16.4)** | **17.2 (11.7, 22.7)** |
| *Vigorous* | 181 | | 11.4 (-6.1, 29.0) | **19.4 (6.9, 31.8)** |
| *p* for equality^d^ |  | |  | 0.38 |
|  |  | |  |  |
| Reference group CPT tolerance**^c^** | 1,962 | | No chronic pain  *99.2 (95.6, 102.8)* | Chronic pain  *99.6 (94.7, 104.5)* |
| LTPA^e^ |  | |  |  |
| *Sedentary* | 1,962 | | 0 (reference) | 0 (reference) |
| *Light* | 6,178 | | **7.7 (3.7, 11.8)** | 4.8 (-0.7, 10.2) |
| *Moderate* | 1,933 | | **13.3 (8.3, 18.2)** | **17.1 (9.6, 24.5)** |
| *Vigorous* | 181 | | **14.2 (2.8, 25.6)** | **26.2 (3.5, 49.0)** |
| *p* for equality^d^ |  | |  | 0.21 |
| **^a^** Mixed model Tobit regression with upper limit (censoring)=106 s. for latent distribution of CPT outcome. Models were adjusted for measurement occasion, as well as baseline sex, age, and self-reported occupational PA level, education, alcohol consumption frequency, smoking status, health status, and chronic pain. Significant results in **bold**.  **^b^** Modelling with interactions LTPA∙sex or LTPA∙chronic pain. **^c^** Model-predicted sedentary CPT tolerance at means of covariates. ^d^ Test of interaction between LTPA and sex or chronic pain using the likelihood ratio test.  **^e^** Model additionally adjusted for chronic pain. Abbreviations: LTPA=leisure-time physical activity; CPT=cold pressor test. | | | | |
