# Supplementary material for: Longitudinal relationships between habitual physical activity and pain tolerance in the general population
Source: PLoS One. 2023 May 24;18(5):e0285041. doi: 10.1371/journal.pone.0285041 (PMC10208467; doi:10.1371/journal.pone.0285041)
Supplement: S4 Table — The Tromsø Study 2007–2016. Censored estimates (all censored values included as is) by linear mixed models with random intercept. Models were adjusted for measurement occasion, as well as baseline sex, age, and self-reported occupational PA level, education, alcohol consumption frequency, smoking status, health status, and chronic pain. Significant results in bold. (DOCX) [file pone.0285041.s004.docx]

**Table S4: Regression coefficients with 95% confidence limits for the association between baseline levels of leisure-time physical activity and cold-pressor tolerance time (seconds) according to sensitivity analyses. The Tromsø Study 2007-2016.**

|  |  | Overall effect |
| --- | --- | --- |
| **LTPA** | **n=10,254** |  |
| Censored CPT^a^ |  |  |
| *Sedentary* |  | *0 (reference)* |
| *Light* |  | ***3.1 (1.6, 4.6)*** |
| *Moderate* |  | ***6.4 (4.5, 8.3)*** |
| *Vigorous* |  | ***6.7 (2.2, 11.1)*** |

^a^ Censored estimates (all censored values included as is) by linear mixed models with random intercept.
Models were adjusted for measurement occasion, as well as baseline sex, age, and self-reported occupational PA level, education, alcohol consumption frequency, smoking status, health status, and chronic pain. Significant results in **bold**.
CPT=cold pressor test.
