# Supplementary material for: Longitudinal relationships between habitual physical activity and pain tolerance in the general population
Source: PLoS One. 2023 May 24;18(5):e0285041. doi: 10.1371/journal.pone.0285041 (PMC10208467; doi:10.1371/journal.pone.0285041)
Supplement: Attachment — Submitted filename: Response to reviewers.docx [file pone.0285041.s005.docx]

Dear Editor,

Thank you for the continued opportunity to submit our article to PLOS ONE, and for the sensible and thorough feedback provided. Within you will find updated marked-up and track changes-free manuscripts that address the excellent suggestions provided by your editorial team and the reviewers. We are grateful for the opportunity to improve our manuscript and for the collaboration in ensuring this study will be of the highest quality once published. Below are included the detailed rebuttals for each suggested improvement, including explanations on where to find these changes in the attached manuscripts. Finally, all figures have been updated using the Preflight Analysis and Conversion Engine, and re-submitted as requested.

We look forward to hearing from you and the reviewers again, and hope this updated manuscript remains interesting for publication in your journal.

Sincerely

Anders P. Årnes (on behalf of the authors)

Editorial review rebuttals:

1. Some minor revision was done to the formatting of references to tables and figures throughout the text to better reflect the journal’s format requirements. Likewise, author formatting and contributions were updated on title page and author contributions were hence removed from the end of the manuscript body.
2. Regarding the Data Availability Statement: The data for this study cannot be shared publicly because the current study is based on data owned by a third party (The Tromsø Study, Department of Community Medicine, UiT The Arctic University of Norway). Ethical and legal restrictions prevent data from being made publicly available. These revolve around the protection of potentially sensitive data that cannot be shared publicly without being in risk of breaching data protection laws. Bona fide researchers can apply for data from the Tromsø Study. Guidelines on how to access the data are available at the website https://uit.no/research/tromsostudy. All inquiries about the Tromsø Study should be sent by e-mail to [tromsous@uit.no](mailto:tromsous@uit.no). Similar reasoning has been given for previous publications of Tromsø Study-based studies in PLOS ONE.
3. The reference list has been checked for incorrect citations. Citation 29 was faulty and has been replaced with the correct citation. Citation 43 appears to no longer be available online and so has been removed. The description of the statistical package remains as a part of the package documentation.

Reviewer rebuttals:

3. Please see rebuttal 2 in editorial review rebuttals.

5.
Reviewer #1:
We thank reviewer 1 for a kind appraisal and sensible suggestions for improvement. We are also very much in agreement with the reviewer on the possible benefit of adding more measurement occasions to this longitudinal study. This would allow for some additional longitudinal designs that could be useful in assessing changes over time. At the moment, these are the only two repeated surveys (in the world) that have included population-level assessment of quantitative sensory measurements of experimental pain sensitivity and so this has not been possible. However, Tromsø8 is scheduled to start in 2025 approximately, and the Tromsø Pain Study research group has applied to have the QST-measurement station included in this survey too, which would add an additional measurement occasion to this longitudinal data set and facilitate further longitudinal studies.

In the revised manuscript, we have attempted to improve the flow and readability of the discussion section using single-sentence revisions, paragraph partitioning, and adding or changing connecting clauses. Some clauses were also relocated to more suitable positions in the body of text. Finally, we changed the final discussion heading to ‘Possible limitations’ as this better reflects the following format of consecutively postulating and rebutting limiting factors rather than a strict two-fold division of the sub-chapter.

Reviewer #2:
We thank reviewer 2 for the kind feedback and shared understanding of the relevancy of this research. We have moved the ethics-section as suggested, and done a thorough re-reading of the manuscript body to account for typos. This has resulted in some correction of upper-lowercase typos, a harmonization of descriptions in the statistical analyses-section and tables, and some word or sentence revisions throughout the text for improved readability.
